# Out-of-equilibrium quantum magnetism and thermalization in a spin-3 many-body dipolar lattice system

**DOI:** 10.1038/s41467-019-09699-5

**Published:** 2019-04-12

**Authors:** S. Lepoutre, J. Schachenmayer, L. Gabardos, B. Zhu, B. Naylor, E. Maréchal, O. Gorceix, A. M. Rey, L. Vernac, B. Laburthe-Tolra

**Affiliations:** 1 Université Paris 13, Sorbonne Paris Cité, Laboratoire de Physique des Lasers, F-93430 Villetaneuse, France; 2 CNRS, UMR 7538, LPL, F-93430 Villetaneuse, France; 3 CNRS, UMR 7504, IPCMS, UMR 7006, ISIS, Université de Strasbourg, Strasbourg, France; 4 JILA, NIST and Department of Physics, University of Colorado, Boulder, 80309 USA; 5 Center for Theory of Quantum Matter, University of Colorado, Boulder, CO 80309 USA; 6 ITAMP, Harvard-Smithsonian Center for Astrophysics, Cambridge, MA 02138 USA

## Abstract

Understanding quantum thermalization through entanglement build up in isolated quantum systems addresses fundamental questions on how unitary dynamics connects to statistical physics. Spin systems made of long-range interacting atoms offer an ideal experimental platform to investigate this question. Here, we study the spin dynamics and approach towards local thermal equilibrium of a macroscopic ensemble of *S* = 3 chromium atoms pinned in a three dimensional optical lattice and prepared in a pure coherent spin state, under the effect of magnetic dipole–dipole interactions. Our isolated system thermalizes under its own dynamics, reaching a steady state consistent with a thermal ensemble with a temperature dictated from the system’s energy. The build up of quantum correlations during the dynamics is supported by comparison with an improved numerical quantum phase-space method. Our observations are consistent with a scenario of quantum thermalization linked to the growth of entanglement entropy.

## Abstract (web summary)

Isolated many-body quantum systems do not thermalize with an external environment but in most cases the internal dynamics leads to the emergence of an effective thermal equilibrium for local degrees of freedom. Here the authors study this behaviour with a realization of a long-range spin model.

## Introduction

Ultra-cold atomic systems featuring long-range interactions are becoming ideal platforms for probing strongly correlated out-of-equilibrium quantum behavior and, in particular, the phenomenon of quantum magnetism, where magnetic moments with quantized energy levels (spins) interact with one another^1–3^. Their appeal stems from the fact that they feature internal levels that can be initialized in pure states and coherently evolved with controllable long-range interactions even under frozen conditions. Recently great advances have been accomplished, but, so far have been mostly limited to small systems (hundreds or fewer particles)^4–12^, or to dilute disordered molecular ensembles^13,14^.

Magnetic quantum dipoles featuring sizable magnetic moments offer unique opportunities since magnetic interactions can directly happen in an enlarged set of low-lying hyperfine Zeeman levels and are not forbidden by parity and time-reversal symmetry as is the case with electric dipoles^15^. They offer untapped opportunities as a quantum resource since *S* > 1/2 spin models have more complexity and cost exponentially more resources to classically simulate^16,17^. In fact, the exploration of the complex non-equilibrium dynamics of dipolar-coupled *S* > 1/2 spin models remains a fascinating territory which only starts to be explored^18,19^.

Here we compare our experimental observations of the seven Zeeman populations of an initial spin coherent state made of *S* = 3 spin particles, with different models. We find that our data compare well with exact short time calculations and semiclassical simulations based on a discrete Monte Carlo sampling in phase space^20–22^. We show that the steady state reached at long times is captured by a statistical ensemble with nonzero thermodynamic entropy, by deriving a simple analytical formula, which compares well to both data and semiclassical simulations. We finally study the growth of entanglement by computing the Renyi entropy associated with a local spin. Our studies confirm a scenario of quantum thermalization as a result of the entanglement accumulated during the dynamics.

## Results

### Realization of an XXZ Heisenberg spin model

**Fig. 1:**
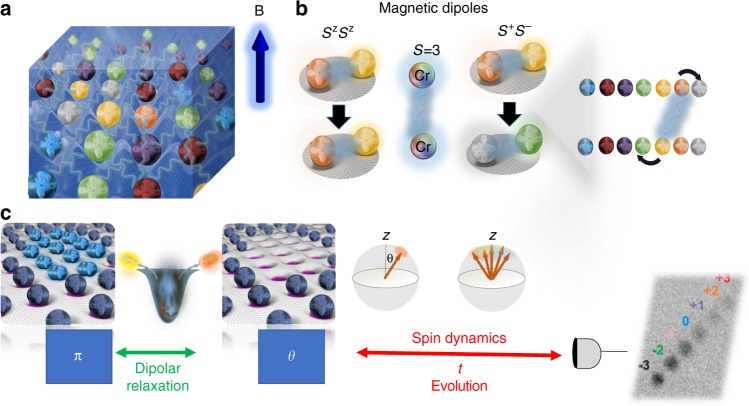
Sketch of the experiment. **a** We consider an assembly of *S* = 3 Cr atoms in an optical lattice prepared in a Mott-insulating state. **b** Dynamics is driven by dipole–dipole interactions which feature both Ising $( {\hat S_i^z\hat S_j^z} )$ and exchange $( {\hat S_i^ + \hat S_j^ - + h.c} )$ terms. **c** A first *π* pulse is used to promote all atoms to the most excited spin state. Dipolar relaxation empties doubly occupied sites. Once this is achieved, a second pulse collectively rotates all spins by an angle *θ* from the external magnetic field **B** which sets the quantization direction. We then study spin dynamics due to intersite dipole–dipole interactions by registering the relative populations of the different Zeeman states

In our system the spin degree of freedom is encoded in the Zeeman levels of the purely electronic *S* = 3 ground state of ^52^Cr atoms. The experiment starts with the production of a spin-3 Bose-Einstein condensate (BEC) of ~4 × 10^4^ atoms in the *m*_*S*_ = −3 state, following the procedure described in ref. ^23^. We then adiabatically load the BEC into a three dimensional (3D) optical lattice made by laser beams at 532 nm^18^. The lattice structure is rectangular in the horizontal plane, and uses a standard retro-reflecting scheme on the vertical axis (see Methods). After loading the atoms into a deep optical lattice, the sample forms a Mott insulator consisting of a core with doubly occupied sites $\left( {\bar n = 2} \right)$, surrounded by a 3D shell of singly occupied sites $\left( {\bar n = 1} \right)$, see Fig. 1.

The experimental procedure to induce spin dynamics is shown in Fig. 1. We initialize the system in a well-characterized state consisting of a macroscopic array of long-lived singly occupied sites close to unit filling, by performing first a filtering protocol. It relies on dipolar relaxation^18^ to empty all doubly occupied sites within the $\bar n = 2$ Mott core after the application of a *π* rf pulse that promotes the atoms to the most energetic spin state *m*_*S*_ = 3. The filtering protocol takes about 7 ms (see Methods). To trigger the spin dynamics we then apply a second rf pulse. This rotates the coherent spin state, such that it forms an angle *θ* with respect to the external magnetic field which sets the quantization axis (see Fig. 1). This prepares a tilted spin coherent state. The spin dynamics is studied by monitoring the time evolution of the population of the different Zeeman states, using absorption imaging after a Stern–Gerlach separation procedure^23^.

A unit-filled array of frozen magnetic dipoles in a lattice interact via dipolar interactions. In the presence of an external magnetic **B** field strong enough to generate Zeeman splittings larger than nearest-neighbor dipolar interactions, only those processes that conserve the total magnetization are energetically allowed and the dynamics is described by the following secular Hamiltonian^18^ (with **B** along the *z* axis):

1 $$\hat H = \mathop {\sum}\limits_{i > j} {V_{ij}} \left[ {\hat S_i^z\hat S_j^z - \frac{1}{2}\left( {\hat S_i^x\hat S_j^x + \hat S_i^y\hat S_j^y} \right)} \right]$$

where the sum runs over all pairs of atoms (*i*,*j*). It corresponds to a XXZ Heisenberg model with dipolar couplings $V_{ij} \equiv \frac{{{\mu} _0(g{\mu} _{\mathrm{B}})^2}}{{4\pi }}\left( {\frac{{1 - 3{\mathrm{cos}}^2{\phi} _{(i,j)}}}{{r_{(i,j)}^3}}} \right)$. Here *μ*_0_ is the magnetic permeability of vacuum, $g \simeq 2$ is the Landé factor, and *μ*_B_ the Bohr magneton. *r*_(*i*,*j*)_ is the distance between atoms, and *ϕ*_(*i*,*j*)_ the angle between their inter-atomic axis and the external magnetic field. The Hamiltonian is given in terms of spin-3 angular momentum operators, $\widehat {\mathbf{S}}_i = \{ \hat S_i^x,\hat S_i^y,\hat S_i^z\}$, associated to atom *i*.

**Fig. 2:**
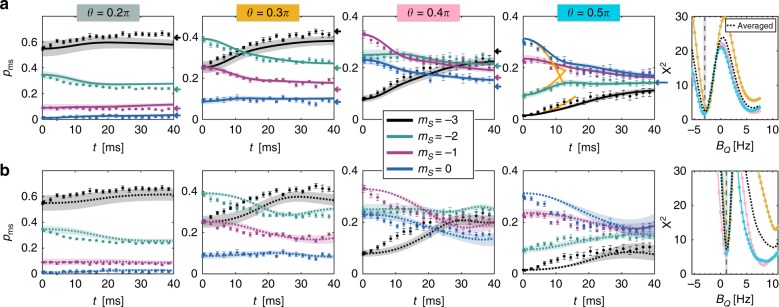
Comparison between classical and quantum dynamics. For various initial tilting angles *θ* = 0.2*π*, 0.3*π*, 0.4*π*, and 0.5*π*, we plot the experimental data for the four lowest spin populations, $p_{m_S}$, and compare them with simulations. **a** Comparison with GDTWA simulations (solid lines) on a 7 × 3 × 7 cluster allowing the quadratic Zeeman field *B*_Q_ to be the only fitting parameter [here: *B*_Q_ = −3.0 Hz]. **b** Comparison with the classical mean-field results (dotted lines) [here: *B*_Q_ = 1.1 Hz]. The two plots on the right show quantitative comparison between data and simulations for *θ* = 0.3*π*, 0.4*π*, and 0.5*π* with a reduced *χ*^2^ criteria, for different values of *B*_Q_. We excluded the *θ* = 0.2*π* case here since it shows no significant dynamics. The best agreement with GDTWA simulations is a factor of three better than with classical simulations. The deviation with the classical simulations is most obvious at short times, and clearly increases with increasing *θ*. The arrows indicate the expected equilibrium population maximizing entropy, for each angle. The orange solid line in panel (**a**) (for *θ* = 0.5*π*) is the result of the perturbative expansion, Eq. (3). The shaded area indicates the range of variation of the populations for evolutions with Δ*B*_Q_ = ±0.3 Hz and uncertainties in the tilting angles with *θ* = (0.2 ± 0.018*π*), (0.3 ± 0.012)*π*, (0.4 ± 0.012)*π*, and (0.5 ± 0.01)*π* (estimated from the experiment). Error bars correspond to statistical standard deviations

An important feature is that the dynamical redistribution of populations can happen for large spins (*S* > 1/2), even though both the total particle number *N* and the collective magnetization $M = \langle \hat S^z\rangle$ are conserved quantities (with $\hat S^{x,y,z} = \mathop {\sum}\nolimits_{j = 1}^N {\hat S_i^{x,y,z}}$). The magnetic dipolar interaction energy between *S* = 3 spins is 36 times larger than the one for *S* = 1/2 alkali atoms, allowing us to probe such population dynamics at milliseconds time scales, as seen in Fig. 2.

### Perturbation theory

We will first introduce the expected basic dynamical features according to time-dependent perturbation theory. We will focus on the main differences when assuming a classical behavior, or when taking into account quantum correlations. The simplest possible picture for the population dynamics relies on a mean-field treatment (i.e., neglecting quantum correlations), where each atom undergoes Larmor precession around an effective dipolar field created by all the other spins, $\hat H^{MF} = \mathop {\sum}\nolimits_{i = 1}^N {{\mathbf{B}}_i^{{\mathrm{eff}}}} \cdot \widehat {\mathbf{S}}_i$, with ${\mathrm{B}}_i^{{\mathrm{eff}}} = - \mathop {\sum}\nolimits_{j = 1}^N \frac{{V_{ij}}}{2}\{ \langle \hat S_j^x\rangle ,\langle \hat S_j^y\rangle , - 2\langle \hat S_j^z\rangle \}$. Time-dependent perturbation theory yields the following equation for $p_{m_S}$, the relative population of Zeeman level *m*_*S*_ = −3, …, 3:

2 $$p_{m_S}^{{\mathrm{MF}}}(t) = p_{m_S}(0) + {\mathrm{sin}}[\theta ]^4\alpha _{m_S}(\theta )t^2{\cal{K}}_{\mathrm{d}}(t) + {\cal{O}}(t^6V_{ij}^6),$$

We give in the Methods section exact formulas for $\alpha _{m_S}(\theta )$. For instance, $\alpha _{m_S = \{ - 3, - 2, - 1,0,1,2,3\} }(\pi /2) = 135/512 \times \{ 1,2, - 1, - 4, - 1,2,1\}$. Here ${\cal{K}}_{\mathrm{d}}(t) \equiv \frac{{t^2}}{{2N}}\mathop {\sum}\nolimits_{i = 1}^N \left[ {\mathop {\sum}\nolimits_{j \ne i}^N V_{ij}B_{ij}^{{\mathrm{dih}}}} \right]^2$. Thus classical dynamics is driven by the dipolar field $B_{ij}^{{\mathrm{dih}}} = - 9/2\mathop {\sum}\nolimits_{k \ne j,i}^N (V_{ki} - V_{kj}){\mathrm{cos}}\theta$. For a homogeneous gas, *B*^dih^ vanishes, and the population dynamics with it. This behavior remains valid at all times given that, by preparation, all spins point along the same direction initially, they precess around the same classical dipolar field, and thus evolve identically. Therefore, the local magnetization $\langle S_i^z\rangle = M/N$ remains constant for each spin, cancelling population dynamics altogether. On the other hand, in a trapped gas, the inhomogeneous dipolar field introduces a differential precession rate between spins, which results in population dynamics. Note that *B*^dih^ is determined by border effects and also that ${\cal{K}}_{\mathrm{d}}(t)$ itself is time dependent (∝*t*^2^). Therefore, classically, population redistribution is a slow *t*^4^ process. We emphasize that *B*^dih^ is proportional to cos *θ* and thus vanishes when *θ* = *π*/2 where no mean-field dynamics takes place at all.

Quantum fluctuations can drastically modify this behavior and induce much faster population dynamics even for a homogeneous gas^24^. Second-order time-dependent perturbation theory on the exact Hamiltonian^25,26^ in Eq. (1) yields:

3 $$p_{m_S}(t) = p_{m_S}(0) + {\mathrm{sin}}[\theta ]^4\alpha _{m_S}(\theta )t^2V_{{\mathrm{eff}}}^2 + {\cal{O}}(t^4V_{ij}^4).$$

In contrast to the mean-field case, the dynamics grows as *t*^2^ and is driven by $V_{{\mathrm{eff}}} \equiv \sqrt {\mathop {\sum}\nolimits_{i,j \ne i}^N V_{ij}^2/N}$. We emphasize the relatively fast decay of $V_{{\mathrm{eff}}}^2$ with interparticle distance *r* (as *r*^−6^), which makes the short time evolution mainly determined by the nearest-neighbor interactions. As *V*_eff_ is independent of *θ* the tipping angle *θ* provides a way to study an out-of-equilibrium magnetism increasingly determined by quantum correlations as *θ* → *π*/2.

In the experiment, external systematics such as quadratic Zeeman fields, *B*_Q_, generated by tensorial light shifts induced by the lattice lasers—with eigenenergies $B_{\mathrm{Q}}m_S^2$—or inhomogeneities associated with magnetic field gradients, Δ_*ij*_ = *B*_*i*_ − *B*_*j*_, need to be accounted for. Their role in the short time dynamics can be understood using perturbation theory (see Methods). Quadratic Zeeman fields can be accounted for by replacing ${\cal{K}}_{\mathrm{d}}(t) \to {\cal{K}}_{\mathrm{d}}(t) - 4/3Q^2$ and $V_{{\mathrm{eff}}}^2 \to V_{{\mathrm{eff}}}^2 - 4/3Q^2$ in the classical and quantum cases, respectively, with $Q^2 \equiv \frac{1}{N}\mathop {\sum}\nolimits_{j \ne i}^N V_{ij}B_{\mathrm{Q}}$. At the mean-field level Δ_*ij*_ directly renormalizes $B_{ij}^{{\mathrm{dih}}} \to B_{ij}^{{\mathrm{dih}}} + {\mathrm{\Delta }}_{ij}$. Thus dipolar inhomogeneities and magnetic field gradients are in direct competition. In the quantum case magnetic field gradients also enter as *t*^4^ but in this case they play a subdominant role since the leading dipolar dynamics is significantly faster (∝*t*^2^).

### Numerical methods to model the spin dynamics

Although perturbation theory allows emphasizing some of the main qualitative differences in the classical and in the quantum regime, to accurately describe the population dynamics we need to go beyond perturbation theory. To accomplish that we parameterize each spin *i* by a generalized Bloch vector, $\vec \lambda ^{[i]}$. In contrast to spin-1/2 systems, this vector is a 48-dimensional object that determines all independent elements of the 7 × 7 (= (2*S* + 1)^2^) individual spin-3 density matrices, $\hat \rho _i(\vec \lambda ^{[i]})$^27^. Inserting the product-state ansatz of the system density matrix, $\hat \rho = \mathop {\prod}\nolimits_{i = 1}^N \hat \rho _i(\vec \lambda ^{[i]})$, into the von-Neumann equation, $d\hat \rho /dt = ( - {\mathrm{i}}/\hbar )[\hat H,\hat \rho ]$, yields *N* × 48 independent non-linear mean-field equations, in which each generalized Bloch vector evolves in the field of the others. The mean-field “classical” results are obtained by numerically integrating these equations of motion (see Methods).

To capture the build up of quantum correlations we developed a generalization of a semiclassical method (generalized discrete truncated Wigner approximation, GDTWA) based on a discrete Monte Carlo sampling in phase space originally derived in the framework of the so-called truncated Wigner approximation (TWA)^28^. It describes the initial state in terms of a probability distribution. Initial spin coherent states are ideal since they can be fully described by a positive discrete probability distribution. For spin-1/2 systems, randomly sampling this initial “Wigner function”, leads to the discrete truncated Wigner approximation (DTWA)^20–22^, an approximation that has been remarkably successful and can capture complex quantum aspects of spin dynamics. In contrast to the spin-1/2 case, here (GDTWA) the discrete probabilities are not provided by the eigenvalues of the three Pauli matrices, but instead by the eigenvalues of the corresponding 48 generalized SU(7) generators (see Methods). This semiclassical approach is benchmarked by comparison with exact diagonalization predictions (see Supplementary Note 1 and Supplementary Fig. 1).

### Comparisons between experiment and numerical simulations

We now describe how our data compare with simulations for different values of *θ*. In Fig. 2, we show our data and the comparisons to both the classical and the GDTWA models. The theoretical models take into account the 3D lattice structure and the measured magnetic field gradients along all three directions. We also include the weak quadratic Zeeman field present in the experiment. Since we could not measure it directly we allow it to be a fitting parameter (see Supplementary Note 2 and Supplementary Fig. 2). For each of the four tilting angles used for the measurements we plot the evolution of fractional populations in different Zeeman states. We only plot the most relevant Zeeman states (the most populated for most of the angles, and only the negative Zeeman states for the symmetric case *π*/2—see Supplementary Fig. 3 for extensive data). Experimentally, we find that the amplitude of spin dynamics (i.e., the amplitude of the variations of the populations in the different spin states) is stronger when the angle increases.

**Fig. 3:**
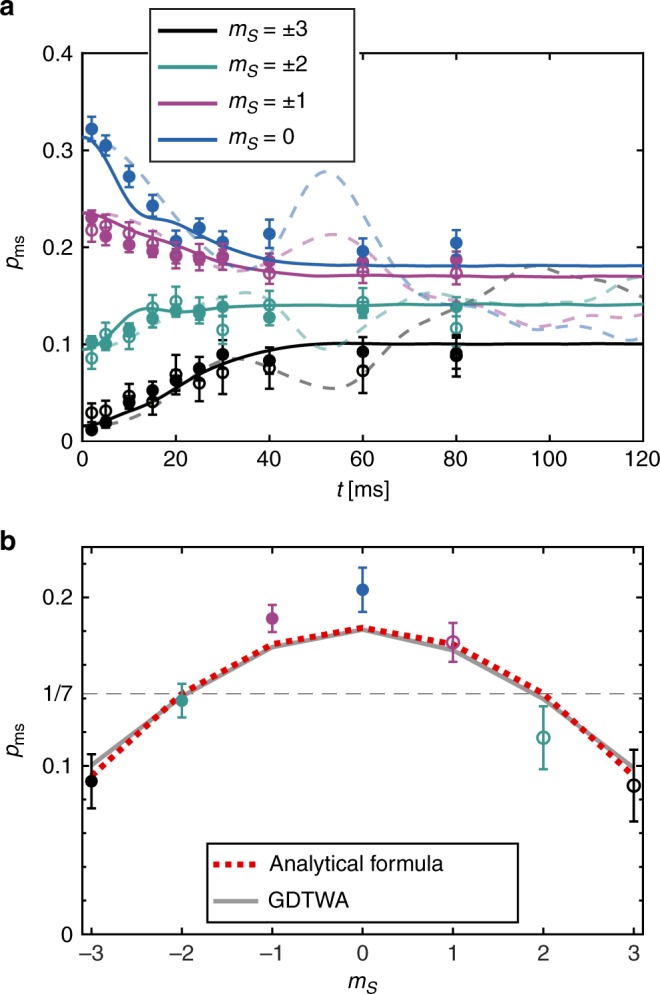
Quantum thermalization. **a** Long-time evolution of state populations for *θ* = *π*/2: Long-time experimental data points are compared with corresponding best fitting GDTWA (solid lines, *B*_Q_ ≈ −3.6 Hz) and mean-field (thin dashed lines, *B*_Q_ ≈ 1.1 Hz) simulations. **b** The experimental data points at *t* = 80 ms are compared with the GDTWA prediction (including field gradients), and the analytical quantum thermalization expression, Eq. (6), which corresponds to an effective temperature of −2.5 nK, using *B*_Q_ = −3.6 Hz. Error bars correspond to statistical standard deviations

At small angles the experimental data are qualitatively reproduced by both classical and GDTWA simulations. As can be seen in Fig. 2, both simulations then yield similar results, but nevertheless show systematic differences. This shows that even at the smallest angles that we have probed, beyond mean-field effects are in principle already at play. However, given the signal-to-noise ratio in the experimental data, it is difficult to quantify the contribution of beyond mean-field effects to the spin dynamics at weak rotations. When increasing the angle, it becomes increasingly clear that only the beyond-mean-field simulation accounts for the observed dynamics, both at short times (*t* < 20 ms, see Fig. 2) and at long times (*t* > 40 ms, see Fig. 3a).

We have performed a systematic study in our numerical simulations, by varying the size of the system which we use. We find that a good agreement between experiment and beyond mean-field theory is only reached, provided the number of interacting spins in the simulation is larger than about 60 (see Supplementary Note 4 and Supplementary Fig. 4). Taken together, these data show that spin dynamics after the initial quench is inherently many-body, and beyond the grasp of mean-field models. As can also be seen in Fig. 2, our experimental data at short times are also in excellent agreement with the exact dynamics calculated within the framework of second-order time-dependent perturbation theory (see Eq. (3)). Also in good agreement with this equation, we find the dynamics at short times to be roughly independent of the magnetic field gradient applied to the sample (up to values >30 MHz m^−1^; see Supplementary Note 5 and Supplementary Fig. 5). In contrast, we point out that the experimental data at short times systematically show faster dynamics than predicted in the classical picture, whose initial *t*^4^ dependence (see Eq. (2)) fails to reproduce the experimental observations. Finally, we checked that the effect of imperfections in the lattice preparation on GDTWA predictions is small (see Supplementary Note 6 and Supplementary Fig. 6).

### Models for thermalization at long times

For an isolated system, entanglement build up after a quench into a non-equilibrium situation is tied to the scenario of quantum thermalization. To support the relevance of quantum correlations during dynamics, we thus analyze the long-time behavior of the populations. For all tipping angles *θ*, we observe that the experimental system approaches a steady state, which is in agreement with predictions of closed system quantum thermalization, given, e.g., by the eigenstate thermalization hypothesis (ETH)^29,30^. In particular, we find that the long-time average populations are very well described by the effective thermal distribution $\hat \rho _{{\mathrm{cT}}}(\beta ,\mu ) = \frac{{e^{ - \beta \hat H_{\mathrm{T}} - \mu \hat S^z}}}{{{\mathrm{tr}}[e^{ - \beta \hat H_{\mathrm{T}} - \mu {\hat S}^z}]}}$ where the chemical potential *μ* and inverse temperature *β* = 1/*k*_B_*T* are set by the energy and magnetization of the initial pure state:

4 $$\left\langle {\hat H_{\mathrm{T}}} \right\rangle = {\mathrm{tr}}\left[ {\hat \rho _{{\mathrm{cT}}}\left( {\beta ,\mu } \right)\hat H_{\mathrm{T}}} \right]\quad \left\langle {\hat S^z} \right\rangle = {\mathrm{tr}}\left[ {\hat \rho _{{\mathrm{cT}}}\left( {\beta ,\mu } \right)\hat S^z} \right],$$

which are conserved throughout the evolution. Here $\hat H_{\mathrm{T}} = \hat H + \mathop {\sum}\nolimits_i B_{\mathrm{Q}}(\hat S_i^z)^2$ is the total Hamiltonian. As shown in Fig. 2, the steady-state populations approach the ones (indicated by the arrows for all tipping angles) dictated by the thermal ensemble when simply setting *β* = 0 (in which case the maximum-entropy state only depends on magnetization). For angles close to *π*/2, however, where quantum effects are most significant, we find a deviation compared with this simplistic prediction. We therefore proceed to study this interesting regime.

In Fig. 3 we show dynamics up to longer times, and confirm that a steady state is indeed reached after 40 ms for the *π*/2 case, a feature that the GDTWA simulation reproduces, while classical simulations predict an oscillatory behavior for a much longer duration. This qualitative difference between the classical and the quantum behavior is associated with the different origin of thermalization in both pictures: while quantum-mechanically thermalization is tied to the growth of entanglement, classically, reaching a steady state in a system of frozen particles is a consequence of the single-particle dephasing induced by field inhomogeneities. The inhomogeneous fields arise either from external fields, or from effective fields generated on one particle from the mean-field interactions with the surrounding particles. This behavior differs from the typical thermalization scenario in mobile particles where collisions can classically change both motional and internal degrees of freedom while redistributing energies and momenta^29,31–33^. Our observations clearly rule out a simple mean-field (classical) behavior. Most interestingly, we find a very good agreement between the experimental data points taken at long times, i.e. after the system has reached its steady state, if instead of setting *β* = 0 we account for the corrections generated by the quadratic Zeeman field *B*_Q_, and the finite but small energy of the initial state in Eq. (4). Using a simple perturbative approach (see Methods) we obtain that the *β*^(0)^ = *μ*^(0)^ = 0 solutions should be replaced by

5 $${\beta ^{(1)} = \frac{{{\mathrm{tr}}[\hat \rho _{{\mathrm{cT}}}(0,0)\hat H_{\mathrm{T}}] - \langle \hat H_{\mathrm{T}}\rangle }}{{{\mathrm{tr}}[\hat \rho _{{\mathrm{cT}}}(0,0)\hat H_{\mathrm{T}}^2] - {\mathrm{tr}}[\hat \rho _{{\mathrm{cT}}}(0,0)\hat H_{\mathrm{T}}]^2}} = \frac{{5B_{\mathrm{Q}} + 9\bar V}}{{24V_{{\mathrm{eff}}}^2 + 24B_{\mathrm{Q}}^2}}\quad \quad \mu ^{(1)} = 0}$$

leading to:

6 $$p_{m_S}(t_{{\mathrm{SS}}}) \approx {\mathrm{tr}}[\hat \rho _{{\mathrm{cT}}}(\beta ^{(1)},0)\hat p_{m_S}] = \frac{1}{7}(1 - \beta ^{(1)}B_{\mathrm{Q}}(m_S^2 - 4)),$$

with $\bar V \equiv 1/N\mathop {\sum}\nolimits_{i > j} V_{ij}$. We find $\bar V/h \approx - 0.57$ Hz and $V_{{\mathrm{eff}}}/h \approx 6.13\,{\mathrm{Hz}}$ for our lattice geometry. As $\bar V < 0$, negative temperatures are expected for low enough *B*_Q_ (as allowed for a system whose maximum energy is bounded). Figure 3b shows a very good agreement between the equilibrium data and the analytical model (see Supplementary Note 7 and Supplementary Fig. 8 for extensive equilibrium data). For this comparison, there is no free parameter, since we use the value of *B*_Q_ for which the dynamical evolution of the spins is best reproduced by GDTWA simulations. This good agreement confirms the scenario that the coherent Hamiltonian evolution of the many-body system drives it toward a strongly entangled pure state for which the observables display thermal-like behavior. The agreement between the analytical model and the GDTWA at long times shown in Fig. 3b also indicates that the GDTWA not only captures the short term dynamics (as previously known from the theoretical point of view), but also the approach to equilibrium.

To compare the analytical formula to the data we have ignored magnetic field gradients in Eq. (6) (See Methods). In principle, magnetic field gradients should lead to an equilibrium state where a spatial texture of magnetization develops. However, such a texture requires long-range interactions between remote parts of the cloud, which only occurs for an extremely long timescale for dipolar interactions. We have verified (see Supplementary Note 7 and Supplementary Fig. 7) that indeed magnetic field gradients can be neglected to evaluate the quasi-steady state populations reached at 100 ms. This shows that a local equilibrium is first reached, well before the full many-body system may reach true equilibrium with maximum entropy, where all populations in the different Zeeman states would be equally populated.

### Study of quantum correlations

To quantify the importance of quantum correlations in the spin dynamics as a function of the tilting angle *θ*, we analyze from the theoretical point of view the properties of the reduced density matrix for each spin, $\hat \rho _i(\vec \lambda ^{[i]})$. In our simulations those density matrices are readily available from the generalized Bloch vectors. To minimize finite size and boundary effects we focus on the density matrix of the central spin of our simulated block $\hat \rho _0$. Even when, as in our simulations, the quantum state of the full system $\hat \rho$ is pure, the reduced single-spin density matrices can assume a mixed character due to the buildup of entanglement between the spins. This mixed character is quantified by a reduced purity, ${\mathrm{t}}r(\hat \rho _0^2) \, < \, 1$ and thus an increased entropy, which we compute in terms of the second-order Renyi entropy, $S_0^{(2)} = - {\mathrm{log}}_2[{\mathrm{tr}}(\hat \rho _0^2)]$. If the state of the full system is pure, ${\mathrm{t}}r(\hat \rho ^2) = 1$, the Renyi entropy is a measure of entanglement^34^: it is zero for product states, and reaches the maximum value of $S_0^{(2){\mathrm{max}}} = {\mathrm{log}}_2[7]$ (the value for a fully mixed state of a spin-3 particle) for many-body states where the quantum information encoded in an individual spin is completely scrambled due to entanglement with other ones.

**Fig. 4:**
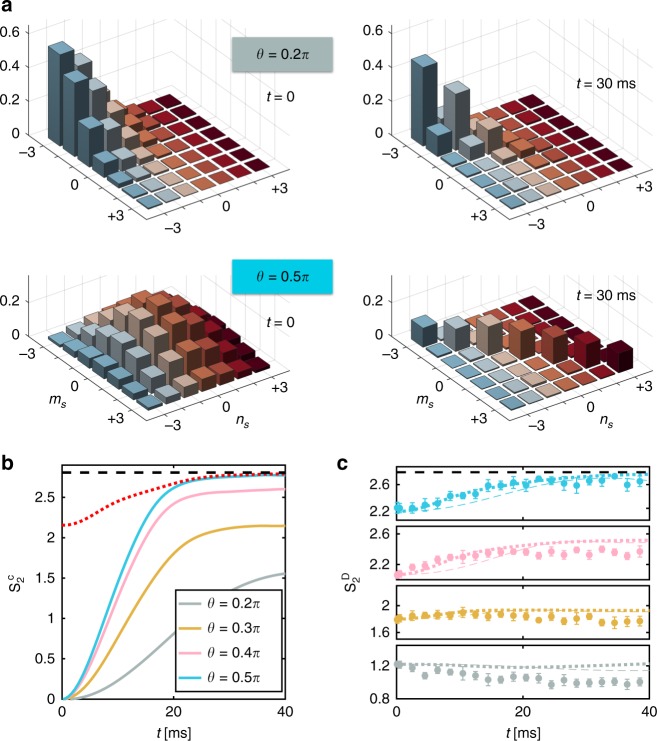
Entanglement buildup. **a** Absolute values of the central spin density-matrix elements $|\rho _{m_S,n_S}^{\mathrm{c}}|$ with $\rho _{m_s,n_S}^{\mathrm{c}} \equiv \langle m_S|\hat \rho _0|n_S\rangle$, extracted from GDTWA simulations with the same parameters as in Fig. 2. Off-diagonal single-site coherences are destroyed as the spins become entangled during the quantum dynamics (left two panels: *t* = 0, after the tilt, right two panels: after *t* = 30 ms evolution). For small rotation angles (upper panels: *θ* = 0.2*π*), the system evolves locally into a partially mixed state (uneven spin-state population). For larger rotation angles (lower panels: *θ* = 0.5*π*), the local state resembles a maximally mixed state of the form $\hat \rho _0 \propto \frac{1}{7}\mathop {\sum}\nolimits_{m_S = - 3}^3 |m_S\rangle \langle m_S\rangle$. **b** Evolution of $S_2^{\mathrm{c}}$, the value of the second-order Renyi entropy $S_0^{(2)}$ for the central spin density matrix. For larger rotation angles, the entanglement entropy increases with time, almost reaching the maximum value ($S_0^{(2){\mathrm{max}}} = {\mathrm{log}}_2(7)$, black dashed line). The red dotted line shows the upper bound $S_2^{\mathrm{D}}$, computed only from the diagonal elements (populations) of the average single-site density matrix $\hat \rho _S$ (see text) for *θ* = 0.5*π*. **c** Comparison of the theoretically computed diagonal entropy (GDTWA: thick dotted line; mean-field: thin dashed line) with the one reconstructed from the measured populations. Error bars correspond to statistical standard deviations

As can be seen in Fig. 4, the quantum evolution leads to a growth of $S_0^{(2)}$ already for the smallest investigated angle *θ* = 0.2*π*. The dynamical growth of entanglement increases significantly for larger tilt angles. At *θ* = *π*/2, we find that $S_0^{(2)}$ approaches its maximum possible value $S_0^{(2){\mathrm{max}}}$. Although we cannot perform a full state-tomography from the experimental data, we can compare our experimental data to a “diagonal entropy” computed in terms of the diagonal part of the averaged single-particle density matrix $\hat \rho _{\mathrm{S}} = (1/N)\mathop {\sum}\nolimits_{i = 1}^N \hat \rho _i(\vec \lambda ^{[i]})$. Note that for an homogeneous system $\hat \rho _{\mathrm{S}} = \hat \rho _0$. In this case we can define this entropy as $S_2^{\mathrm{D}} = - {\mathrm{log}}_2\{ {\mathrm{tr}}[{\mathrm{diag}}(\hat \rho _{\mathrm{S}})^2]\}$, which can be readily accessed from the population data, assuming homogeneity: $S_2^{\mathrm{D}} = - {\mathrm{log}}_2\{ {{\sum} p_{m_S}^2} \}$.

This diagonal entropy is not an entanglement witness, but it provides an upper bound of the entanglement entropy, $S_2^{\mathrm{D}} \ge S_0^{(2)}$. In a translationally invariant system it increases as quantum correlations build up with time and approaches the full entropy as the single-spin density matrices decohere due to entanglement. However, in our finite system, boundary effects can obscure this behavior. For example, at small angles, diagonal entropy shows a slight reduction as a function of time (see Fig. 4 for *θ* ≤ 0.3*π*). On the other hand, we do observe it increases with time as the system thermalizes for large values of *θ*, in which case the non-trivial growth of the experimental diagonal entropy is in excellent agreement with our theoretical estimates, provided quantum fluctuations are taken into account. Moreover it also approaches $S_0^{(2)}$ for *θ* = *π*/2.

## Discussion

In summary, our study demonstrates the dominant role of quantum correlations in the out-of-equilibrium dynamics of an initially uncorrelated spin coherent state, when the angle it makes with the external magnetic field is close to *π*/2. We have shown that our long-range interacting many-particle isolated spin system internally thermalizes through entanglement buildup, and develops an effective thermal-like behavior through a mechanism which is purely quantum and conservative. The comparison between experiment and theory shows that the GDTWA simulations can be trusted for studying the dynamics in a complex quantum many-body system, provided a sufficient number of atoms is included in the simulation. Thus, our experiment provides a test-bed for a theoretical method based on the GDTWA, for systems of large spins, and in a many-body regime where simulations based on exact diagonalization techniques are intractable with current computational resources. In turn, our study can be used as a benchmark of a quantum simulator of the spin-3 XXZ Heisenberg model and opens a path toward the study of open problems in quantum many-body physics. For example, by operating the experiment at smaller lattice depths, where tunneling is allowed, we will have the exciting opportunity to study itinerant magnetism, whose description is typically unaccessible to theory, but which is believed to be at the heart of the physics behind high-temperature superconductivity^35^.

## Methods

### Description of the 3D lattice

The 3D lattice is made with five laser beams at 532 nm. On the horizontal plane, three beams with the same frequency define a rectangular pattern, with respective directions ${\mathbf{u}}_{{\mathbf{H}}_{\mathbf{1}}} = {\mathrm{cos}}(\alpha ){\mathbf{u}}_{\mathbf{x}} - {\mathrm{sin}}(\alpha ){\mathbf{u}}_{\mathbf{y}}$, ${\mathbf{u}}_{{\mathbf{H}}_{\mathbf{2}}} = - {\mathbf{u}}_{{\mathbf{H}}_{\mathbf{1}}}$, ${\mathbf{u}}_{{\mathbf{H}}_{\mathbf{3}}} = {\mathrm{cos}}(\pi /4){\mathbf{u}}_{\mathbf{x}} + {\mathrm{sin}}(\pi /4){\mathbf{u}}_{\mathbf{y}}$, *α* = 8/180*π*. Two other beams, contra-propagating, with a frequency offset by 30 MHz compared with the beams in the horizontal plane, with directions ${\mathbf{u}}_{{\mathrm{V}}_{\mathbf{1}}} = - {\mathrm{cos}}(\beta ){\mathbf{u}}_{\mathbf{z}} + {\mathrm{sin}}(\beta ){\mathbf{u}}_{\mathbf{x}} = - {\mathbf{u}}_{{\mathrm{V}}_{\mathbf{2}}}$, *β* = 7/180*π*, form an independent light pattern. Calibration of the lattice is performed by standard matter wave diffraction pattern analysis after pulsing lattice beams onto the BEC, with the three pairs of beams (**H**_**1**_, **H**_**2**_), (**H**_**1**_, **H**_**3**_), and (**V**_**1**_, **V**_**2**_). The laser powers are chosen so that these three couples of beams induce almost equal lattice depths, larger than 25 recoil energy. For these lattice depths, the tunneling time is typically 100 ms, and tunneling events can safely be neglected during dynamics.

### Preparation of a lattice with only singly occupied sites

To prepare a lattice of atoms at unit filling, we first slowly load the BEC into a 3D optical lattice, to reach a Mott-insulating state. For our experimental parameters, there exists a core with only doubly occupied sites, surrounded by a 3D shell of atoms at unit filling. We empty the doubly occupied sites by performing a rf pulse to promote all atoms from the lowest energy Zeeman state *m*_*s*_ = −3 into the state *m*_*s*_ = 3, which triggers dipolar relaxation. We perform our experiment in presence of an external magnetic field which is large enough that dipolar relaxation can be considered as a short-range process^36^. Thus, only atoms in doubly occupied sites undergo dipolar relaxation, and each dipolar relaxation event empties one doubly occupied lattice site. We estimate the probability of secondary collisions during this filtering procedure to be below 0.05. After 7 ms, all doubly occupied sites are empty, with about 10,000 remaining atoms.

The spin dynamics experiment is then performed using the atoms remaining in the shell with unit occupancy. Because the sample during dynamics consists of a 3D shell of atoms with unit occupancy within the lattice, border effects might not be fully negligible during dynamics. Indeed, our estimates is that about 20 percent of the atoms within the shell of singly occupied sites are close to the boundary. It is likely that spin dynamics is slower for these atoms lying close to the frontier of the shell.

Note that the experiment could not be performed at arbitrarily high magnetic field intensities. As a consequence, some of the atoms which underwent dipolar relaxation remain trapped in very highly excited states of the combined lattice-dipole trap potentials. This translates into losses affecting the sample with unit filling. After 40 ms, from 20 to up to 40 percent of the atoms are typically missing, depending on the magnetic field strength. This phenomenon does not seem to impact the agreement of our spin dynamics data with GDTWA theory as long as losses are below 30 percent.

### Atom number calibration

The number of atoms in different spin states is estimated using standard absorption imaging, after spin separation using an applied magnetic field gradient during the free fall of atoms, following a Stern–Gerlach procedure. The cross section for absorption of resonant light strongly depends on the *m*_*s*_ states, through Clebsch–Gordan coefficients. Therefore, we calibrate the relative sensitivity of the imaging system for the different spin states by comparing the measured populations just after the rf pulse to the theoretically expected values. This calibration depends on the external magnetic field direction during spin dynamics, as eddy currents do not allow to rapidly set its direction during imaging.

For the specific case of *θ* = *π*/2, we employ a slightly different method to calibrate the different sensitivities. Indeed, the number of atoms in *m*_*s*_ = +3 is then very small just after the rf pulse and the detectivity of this Zeeman state is the lowest, due to unfavorable Clebsch–Gordan coefficients. For this specific dataset, we thus enforce that the *m*_*s*_ = −3 and *m*_*s*_ = 3 average atom number after spin dynamics is identical. This choice is motivated by the fact that the Hamiltonian preserves magnetization (as experimentally verified for all other datasets), and by the initially symmetric theoretical populations in the different Zeeman states. For example, for the *π*/2 data in Fig. 2 of the main article, the detectivity correction factors of the different Zeeman states are *f*_−3_ = 0.76, *f*_−2_ = 0.96, *f*_−1_ = 1.18, *f*_0_ = 1.57, *f*_1_ = 2.93, *f*_2_ = 2.68, and *f*_3_ = 5.32.

### Short-time analysis of population dynamics

Using time-dependent perturbation theory we analyze the contribution of the different terms in the Hamiltonian at short times.

For our system the initial population is given by $$p_{m_S}(0) = \left( {\begin{array}{*{20}{c}} 6 \\ {m_S + 3} \end{array}} \right)\left( {{\mathrm{sin}}\left( {\frac{\theta }{2}} \right)} \right)^{(6 + 2m_S)}\left( {{\mathrm{cos}}\left( {\frac{\theta }{2}} \right)} \right)^{(6 - 2m_S)}$$ and the coefficients $\alpha _{m_S}(\theta )$ given by $\alpha _{ - 3}(\theta ) = \frac{{135}}{{32}}{\mathrm{cos}}^8\left( {\frac{\theta }{2}} \right)$, $\alpha _{ - 2}(\theta ) = \frac{{135}}{{32}}{\mathrm{cos}}^6\left( {\frac{\theta }{2}} \right)[1 - 3{\mathrm{cos}}(\theta )]$, $\alpha _{ - 1}(\theta ) = \frac{{135}}{{256}}{\mathrm{cos}}^4\left( {\frac{\theta }{2}} \right)[13 - 20{\mathrm{cos}}(\theta ) + 15{\mathrm{cos}}(2\theta )]$, $\alpha _0(\theta ) = \frac{{135}}{{256}}{\mathrm{sin}}^2(\theta )[3 + 5{\mathrm{cos}}(2\theta )]$, $\alpha _1(\theta ) = \frac{{135}}{{256}}{\mathrm{sin}}^4\left( {\frac{\theta }{2}} \right)[13 + 20{\mathrm{cos}}(\theta ) + 15{\mathrm{cos}}(2\theta )]$, $\alpha _2(\theta ) = \frac{{135}}{{32}}{\mathrm{sin}}^6\left( {\frac{\theta }{2}} \right)[1 + 3{\mathrm{cos}}(\theta )]$, $\alpha _3(\theta ) = \frac{{135}}{{32}}{\mathrm{sin}}^8\left( {\frac{\theta }{2}} \right)$.

### Generalized Bloch vectors and the GDTWA

A generic density matrix for a discrete system with *D* states on site *i* takes the form $\hat \rho _i = \mathop {\sum}\nolimits_{\alpha = 1,\beta = 1}^D c_{\alpha ,\beta }|\alpha \rangle \langle \beta |$. For a spin-3 atom *D* = 7, and to the states $\left| {\alpha = 1,2,3, \ldots ,6,7} \right\rangle$ we may associate the spin states $\left| {m_S = 3,2 \ldots , - 2, - 3} \right\rangle$. Since $(\hat \rho _i)^\dagger = \hat \rho _i$ and ${\mathrm{tr}}(\hat \rho _i) = 1$ a total of *D*^2^ − 1 real numbers are needed to describe an arbitrary state. Those numbers can be expressed as expectation values of *D*^2^ − 1 orthogonal observables: $\hat \Lambda _{\alpha ,\beta < \alpha }^{[i],R} = (|\beta \rangle \langle \alpha | + |\alpha \rangle \langle \beta |)$ and $\hat \Lambda _{\alpha ,\beta < \alpha }^{[i],I} = - {\mathrm{i}}(|\beta \rangle \langle \alpha | - |\alpha \rangle \langle \beta |)$ for 1 ≤ *α* ≤ *D*, 1 ≤ *β* ≤ *D* − 1, and $\hat \Lambda _\alpha ^{[i],D} = \sqrt {\frac{2}{{\alpha (\alpha + 1)}}} \left( {\mathop {\sum}\nolimits_{\beta = 1}^\alpha |\beta \rangle \langle \beta | - \alpha |\alpha + 1\rangle \langle \alpha + 1|} \right)$ for $1 \le \alpha < D - 1$. Here, the $\hat \Lambda _{\alpha ,\beta < \alpha }^{[i],R/I}$ correspond to measurements of the real (“*R*”) and imaginary (“*I*”) parts of the off-diagonal parts of $c_{\alpha ,\beta }$, and $\hat \Lambda _\alpha ^{[i],D}$ to linear combinations of the real diagonal elements $c_{\alpha ,\alpha }$. Together, the set of matrices $\hat \Lambda _\mu ^{[i]} \in \{ \Lambda _{\alpha ,\beta }^{[i],R/I},\hat \Lambda _\alpha ^{[i],D}\}$ are traceless, ${\mathrm{tr}}(\hat \Lambda _\mu ^{[i]}) = 0$ and ${\mathrm{tr}}(\hat \Lambda _\mu ^{[i]}\hat \Lambda _\nu ^{[i]}) = 2\delta _{\mu ,\nu }$. Note that for *D* = 2, the matrices reduce to standard Pauli matrices, for *D* = 3 to standard Gell-Mann matrices. They are known as generalized Gell-Mann matrices (GGMs) and are the generators of the SU(*D*) group^27^.

The mean-field equations can be written as (*D*^2^ − 1) × *N* coupled non-linear equations for the expectation values of $\lambda _\mu ^{[i]} = \langle \hat \Lambda _\mu ^{[i]}\rangle$. The $\lambda _\mu ^{[i]}$ can be interpreted as components of a *D*^2^ − 1 dimensional Bloch vector via the expansion $\hat \rho _i(\lambda _\mu ^{[i]}) = \left[ {{\Bbb I} + \mathop {\sum}\nolimits_{\mu > 0} \lambda _\mu ^{[i]}\hat \Lambda _\mu ^{[i]}} \right]/D$. We denote the Bloch vector elements associated to the off-diagonal and diagonal GGMs as $\lambda _{\alpha ,\beta < \alpha }^{[i],R/I} = (D/2){\kern 1pt} {\mathrm{tr}}(\hat \Lambda _{\alpha ,\beta < \alpha }^{[i],R/I}\hat \rho ^{[i]})$ and $\lambda _\alpha ^{[i],D} = (D/2){\kern 1pt} {\mathrm{tr}}(\hat \Lambda _\alpha ^{[i],D}\hat \rho _i)$, respectively. Furthermore, we define $\hat \Lambda _0^{[i]} = {\Bbb I}\sqrt {2/D}$, such that ${\mathrm{tr}}(\hat \Lambda _0^{[i]}\hat \Lambda _\nu ^{[i]}) = 2\delta _{0,\nu }$. Then, an arbitrary operator can be expanded into the orthogonal basis $\{ \hat \Lambda _\mu ^{[i]}\}$ for 0 ≤ *μ* < *D*^2^. Consider a generic two-spin Hamiltonian between sites *i*, and *j*, and its expansion into GGMs, $\hat H_{i,j} = \mathop {\sum}\nolimits_{\mu ,\nu } h_{\mu ,\nu }^{[i,j]}\hat \Lambda _\mu ^{[i]}\hat \Lambda _\nu ^{[j]}$. Then the mean-field equations of motion follow from inserting a product-state ansatz $\hat \rho = \mathop {\prod}\nolimits_i \hat \rho _i$ into the von-Neumann equations of motion. For the Bloch vector at site *i* (ℏ = 1): $\dot \lambda _\eta ^{[i]} \approx {\kern 1pt} \frac{2}{D}\mathop {\sum}\nolimits_{\mu ,\nu ,\kappa } h_{\mu ,\nu }^{[i,j]}\lambda _\nu ^{[j]}\lambda _\kappa ^{[i]}f_{\mu ,\kappa ,\eta } \equiv \mathop {\sum}\nolimits_\kappa {\cal{F}}_{\eta ,\kappa }^{[i,j]}\lambda _\kappa ^{[i]}$. Here, we defined the “mean-field matrix” ${\cal{F}}_{\eta ,\kappa }^{[i,j]} \equiv \frac{2}{D}\mathop {\sum}\nolimits_{\mu ,\nu } h_{\mu ,\nu }^{[i,j]}\lambda _\nu ^{[j]}f_{\mu ,\kappa ,\eta }$. Here the tensor *f*_*μ*,*κ*,*η*_ is defined via $[\hat \Lambda _\mu ^{[i]},\hat \Lambda _\kappa ^{[i]}] = {\mathrm{i}}{\kern 1pt} f_{\mu ,\kappa ,\eta }\hat \Lambda _\eta ^{[i]}$, whose elements are the structure constants of the SU(*D*) group. The full mean-field equations for the generalized Bloch vector at site *i* are then $\dot \lambda _\eta ^{[i]} = \mathop {\sum}\nolimits_\kappa \left[ {\left( {\mathop {\sum}\nolimits_j {\cal{F}}_{\eta ,\kappa }^{[i,j]}} \right) + h_\kappa ^{[i]}} \right]\lambda _\kappa ^{[i]}$ where $\hat H^{[i]} = \mathop {\sum}\nolimits_\kappa h_\kappa ^{[i]}\hat \Lambda _\kappa ^{[i]}$ is the expansion of the single-site Hamiltonians containing all local terms (field gradients, quadratic Zeeman fields, etc.) into GGMs. It is straightforward to construct the equations for arbitrary Hamiltonians containing single- and two-site terms numerically, as well as to evolve the generalized Bloch vectors in time.

In the numerical mean-field simulations, the quantum state is represented by *N* time-dependent generalized Bloch vectors, $\lambda _\mu ^{[i]}(t)$. We evolve the vectors for the initial state $\mathop {\prod}\nolimits_i |m_S = - 3\rangle _i = \mathop {\prod}\nolimits_i |\alpha = 7\rangle _i$. Explicitly, this state corresponds to a state with $\lambda _{\alpha ,\beta < \alpha }^{[i],R/I}(t = 0) = 0$, $\lambda _{1,2,3,4,5}^{[i],D}(t = 0) = 0$, and $\lambda _6^{[i],D}(t = 0) = - \sqrt {21} = - \sqrt {(D - 1)D/2}$. To also simulate dynamics of initially tilted states, i.e. states created by applying a unitary collective rotation, $\left| {\psi _0} \right\rangle = \mathop {\prod}\nolimits_i \hat U_i(\theta )\left| {m_S = - 3} \right\rangle _i$, we simply rotate the equations of motion by rotating the Hamiltonian ${\hat{H}}\prime = {\mathop {\prod}\limits_i} {{\hat{U}}_i} (\theta ) {\hat{H}} {\mathop {\prod}\limits_j} {\hat{U}}_j^{\dagger} (\theta ) {\hbox{]}}$.

In contrast, in the GDTWA approach we describe the initial state not by a generalized Bloch vector, but instead by a probability “Wigner” distribution, $p_{\mu ,a_\mu }^{[i]}$, for certain discrete configurations of Bloch vector elements, $\lambda _{\mu ,a_\mu }^{[i]}$. Initially, the probabilities and configurations are chosen in such a way that on average $\lambda _\mu ^{[i]}(t = 0) = \mathop {\sum}\nolimits_{a_\mu } p_{\mu ,a_\mu }^{[i]}\lambda _{\mu ,a_\mu }^{[i]} \equiv \overline {\lambda _{\mu ,a_\mu }^{[i]}}$. In practice, the initial multi-spin configurations are selected via a random sampling of $p_{\mu ,a_\mu }^{[i]}$ for each spin *i* and each Bloch vector component *μ*. Then the individually selected configurations are evolved according to the non-linear mean-field equations. Observables are computed from a statistical average over the different trajectories. It is important to note that due to the non-linear nature of the equations, this approach can capture the buildup of correlations, e.g., at later times in general $\overline {\lambda _\mu ^{[i]}\lambda _\nu ^{[j]}(t)} \ne \overline {\lambda _\mu ^{[i]}(t)} {\kern 1pt} \overline {\lambda _\nu ^{[j]}(t)}$.

In particular, as discrete set of initial configurations, $\{ \lambda _{\mu ,a_\mu }^{[i]}\}$, we use a set which is inspired by a “projective measurement, of the GGMs”: for each $\lambda _\mu ^{[i]}(t = 0)$, we choose a set of initial configurations given by the eigenvalues of each GGM. Consider the eigen-expansion of the GGMs, $\hat \Lambda _\mu ^{[i]} = \mathop {\sum}\nolimits_{a_\mu } \eta _{\mu ,a_\mu }^{[i]}\left| {\eta _{\mu ,a_\mu }^{[i]}} \right\rangle \left\langle {\eta _{\mu ,a_\mu }^{[i]}} \right|$, where $\eta _{\mu ,a_\mu }^{[i]}$ and $\left| {\eta _{\mu ,a_\mu }^{[i]}} \right\rangle$ denote the eigenvalues and eigen-vectors, respectively. Then, we choose the “a-th” eigenvalue, $\lambda _\mu ^{[i]}(t = 0) = (D/2){\kern 1pt} \eta _{\mu ,a_\mu }^{[i]}$, with probability $p_{\mu ,a_\mu }^{[i]} = {\mathrm{tr}}\left[ {\hat \rho _0^{[i]}\left| {\eta _{\mu ,a}^{[i]}} \right\rangle \left\langle {\eta _{\mu ,a}^{[i]}} \right|} \right]$, where $\hat \rho _0^{[i]} = \left| {\alpha = 7} \right\rangle \left\langle {\alpha = 7} \right|_i$. Note that this choice is a generalization of the one used for the spin-1/2 DTWA method^20,21^, and for *D* = 2, we reproduce the DTWA sampling. Specifically, for the initial state |*m*_*S*_ = −3〉_*i*_, this prescription leads to fixed “diagonal” Bloch vector elements $\lambda _{1,2,3,4,5}^{[i],D}(t = 0) = 0$ and $\lambda _6^{[i],D}(t = 0) = - \sqrt {21}$, fixed off-diagonal elements $\lambda _{\alpha < 7,\beta < \alpha }^{[i],R/I} = 0$ and fluctuating off-diagonal elements $\lambda _{\alpha = 7,\beta = 1, \ldots 6}^{[i],R/I} \in \{ - D/2, + D/2\}$, each with 50% probability.

### Quantum thermalization

It is generally believed that the unitary quantum evolution of a complex quantum system leads to an apparent maximum-entropy state that can be described by thermodynamical ensembles that properly account for the conserved quantities. In our systems those are the energy and magnetization. We thus postulate that the steady-state properties of local observables, such as the relative population of Zeeman levels, can be described in our system by the thermal distribution $\hat \rho _{{\mathrm{cT}}}(\beta ,\mu ) = \frac{{e^{ - \beta \hat H_{\mathrm{T}} - \mu \hat S^z}}}{{{\mathrm{tr}}[e^{ - \beta \hat H_{\mathrm{T}} - \mu {\hat S}^z}]}}$ where *μ* and *β* = 1/*k*_B_*T* are the chemical potential and inverse temperature set by the energy and magnetization, respectively, accordingly to Eq. (4). While the determination of *β* and *μ* can be a challenging task for a complex many-body system, the anisotropic character of the dipolar interactions facilitates an analytic high-temperature expansion around *β* = 0 since $\bar{V}$ is small (see main text).

Under this assumption, the chemical potential to leading order, is set by $\langle \hat S^z\rangle = {\mathrm{tr}}[\hat \rho _{{\mathrm{cT}}}(0,\mu ^{(0)})\hat S^z] = \frac{{\mathop {\sum}\nolimits_{m_S = - 3}^3 m_Se^{ - \mu ^{(0)}m_S}}}{{\mathop {\sum}\nolimits_{m_S = - 3}^3 e^{ - \mu ^{(0)}m_S}}}$ and therefore $p_{m_S}^{(0)}(t_{{\mathrm{S}}S}) = \frac{{e^{ - \mu ^{(0)}m_S}}}{{\mathop {\sum}\nolimits_{m = - 3}^3 e^{ - \mu ^{(0)}m}}}$. Here *t*_S*S*_ refers to the steady state. These are the populations indicated by arrows in Fig. 2. The case *θ* = *π*/2 is particularly simple since $\langle \hat S_z\rangle = 0$ and thus $\mu ^{(0)} = 0$ and $p_{m_S}^{(0)}(t_{SS}) = 1/7$. This solution, however, shows deviations with the observed long-time dynamics indicating that finite *β* corrections are relevant. To first order in *β*^(1)^ the chemical potential can be written as $\mu ^{(1)} = \mu ^{(0)} + \beta ^{(1)}\delta \nu$ and the solutions of Eq. (4) are described by the relations:

$\langle \hat S^z\rangle = \widetilde {\hat S^z} - \beta ^{(1)}(\delta \nu {\mathrm{\Delta }}\widetilde {\hat S^z\hat S^z} + {\mathrm{\Delta }}\widetilde {\hat H_{\mathrm{T}}\hat S^z})$ and $\langle \hat H_{\mathrm{T}}\rangle = \widetilde {\hat H_{\mathrm{T}}} - \beta ^{(1)}(\delta \nu {\mathrm{\Delta }}\widetilde {\hat H_{\mathrm{T}}\hat S^z} + {\mathrm{\Delta }}\widetilde {\hat H_{\mathrm{T}}\hat H_{\mathrm{T}}})$, where we have defined $\widetilde {\hat {\cal{O}}} \equiv {\mathrm{tr}}[\hat \rho _{{\mathrm{c}}T}(0,\mu ^{(0)})\hat {\cal{O}}]$ and ${\mathrm{\Delta }}\widetilde {\hat {\cal{O}}\hat {\cal{A}}} \equiv \widetilde {\hat {\cal{O}}\hat {\cal{A}}} - \widetilde {\hat {\cal{O}}}\widetilde {\hat {\cal{A}}}$.

Solutions of those equations, taking into account the intial magnetization and total energy in the intial product state, are particularly simple for the *θ* = *π*/2 case, where *μ*^(0)^ = 0, $\widetilde {\hat S}^z = 0$, ${\mathrm{\Delta }}\widetilde {\hat H_{\mathrm{T}}\hat S^z} = 0$ and ${\mathrm{\Delta }}\widetilde {\hat S^z\hat S^z} = NI_2$, $\widetilde {\hat H_T} = NB_{\mathrm{Q}}I_2$ and ${\mathrm{\Delta }}\widetilde {\hat H_{\mathrm{T}}\hat H_{\mathrm{T}}} = N(B_{\mathrm{Q}}^2(I_4 - I_2^2) + 3/4V_{{\mathrm{eff}}}^2I_2^2)$ with $I_r = (\mathop {\sum}\nolimits_{m = - 3}^3 m^r)/7$ (thus *I*_2_ = 4 and *I*_4_ = 28). Those yield the expressions for *β*^(1)^ and *μ*^(1)^ quoted in Eq. (5). In the presence of linear gradients $\hat H_{\mathrm{T}} \to \hat H_{\mathrm{T}} + \mathop {\sum}\nolimits_{i=1}^N B_{i }\hat S_i^z$, under the assumption that $\mathop {\sum}\nolimits_{i= 1}^{^N} B_{i } = 0$, the inverse temperature equation in Eq. (6) for the case of *θ* = *π*/2 should be replaced by $\beta ^{(1)} = \frac{{5B_{\mathrm{Q}} + 9\bar V}}{{24V_{{\mathrm{eff}}}^2 + 24B_{\mathrm{Q}}^2 + 8V_{\mathrm{B}}^2}}$ with $V_{\mathrm{B}}^2 = 1/N\mathop {\sum}\nolimits_{i = 1}^N B_i^2$.

## Supplementary information

Supplementary Information

## Data Availability

The experimental data supporting the findings of this study are available within the paper and its Supplementary Material. Additional numerical data and computer codes used in this study are available from the corresponding author upon request.
