# Supplementary material for: Out-of-equilibrium quantum magnetism and thermalization in a spin-3 many-body dipolar lattice system
Source: Nat Commun. 2019 Apr 12;10:1714. doi: 10.1038/s41467-019-09699-5 (PMC6461643; doi:10.1038/s41467-019-09699-5)
Supplement: Supplementary file 1 — Supplementary Information [file 41467_2019_9699_MOESM1_ESM.pdf]

Supplementary Online Material: Out-of-equilibrium quantum magnetism and thermalization in a spin-3 many-body dipolar lattice system

S. Lepoutre[1,2], J. Schachenmayer[3], L. Gabardos[1,2], B. Zhu[4,5,6], B. Naylor[1,2], E. Maréchal[1,2], O. Gorceix[1,2], A. M. Rey[4,5,7], L. Vernac[1,2], B. Laburthe-Tolra[1,2]

[1]*Université Paris 13, Sorbonne Paris Cité, Laboratoire de Physique des Lasers, F-93430, Villetaneuse, France*

[2]*CNRS, UMR 7538, LPL, F-93430, Villetaneuse, France*

[3]*CNRS, UMR 7504, IPCMS; UMR 7006, ISIS; and Université de Strasbourg, Strasbourg, France*

[4]*JILA, NIST and Department of Physics, University of Colorado, Boulder, USA*

[5]*Center for Theory of Quantum Matter, University of Colorado, Boulder, CO 80309, USA*

[6]*ITAMP, Harvard-Smithsonian Center for Astrophysics, Cambridge, MA 02138, USA*

[7]*Corresponding author: arey@jilau1.coloado.edu*

Supplementary Note 1

Benchmark of the GDTWA on a small plaquette We simulate dynamics of atoms in a 3D lattice geometry. The rapid Hilbert-space growth with system size (7^N) prohibits an exact diagonalization simulation in a real 3D system. The lattice constants in the different dimensions are $(d_x, d_y, d_z) \approx (1.12, 2.24, 1.01) \times \lambda/2$. Due to this geometry and the large spacing along the y-direction, dipole-couplings along the y dimension are relatively small. Thus, to check the validity of our GDTWA simulation, we benchmark it against the exact solution on a small 2D plaquette in the $x - z$ plane. Supplementary Figure 1 shows a benchmark of the GDTWA prediction for the population dynamics for all different tilt angles on a $L_x \times L_y \times L_z = 2 \times 1 \times 4$ plaquette (otherwise the same parameters as in the main text are used). The agreement of the GDTWA (lines) with the ED results (points) is remarkable. Furthermore, we note that modeling the experiment with larger 3D system sizes remains crucial, as is seen by the large difference to the GDTWA result for the large $6 \times 3 \times 6$ system.

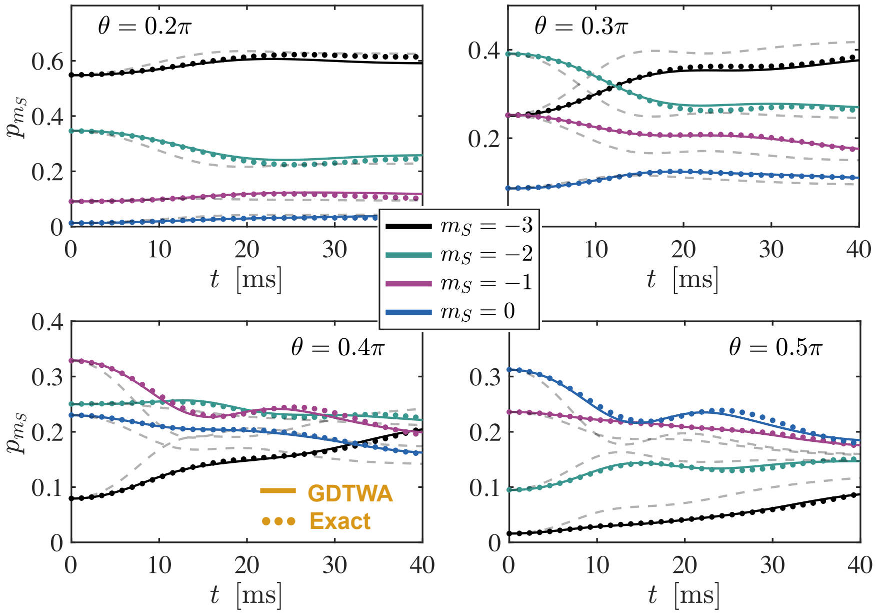

Supplementary Figure 1: Benchmark of the GDTWA on a small plaquette – Time evolution of state populations for all four different initial tilt angles for the parameters used in the main text on a small $L_x \times L_y \times L_z = 2 \times 1 \times 4$ plaquette. The exact-diagonalization results (points) are compared to the GDTWA predictions (lines). The GDTWA provides excellent quantitative predictions on the considered time-scale. As a comparison, the large system GDTWA ($6 \times 3 \times 6$) results are shown as thin dashed grey lines.

Supplementary Note 2

Details on determination of best quadratic shift Here, we provide more details on our determination of the best value of B_{Q}, which we take as only fitting parameter for the full numerical simulations to the experimental data points. We compute the deviation from each experimental data point as $\chi^2_{m_S}(t) = [p^{\text{[sim.]}}_{m_S}(t) - p^{\text{[exp.]}}_{m_S}(t)]^2/\sigma^2_{m_S}(t)$, with $p^{\text{[sim./exp.]}}_{m_S}(t)$ the simulated and experimental state population, respectively and $\sigma_{m_S}(t)$ the experimental error bar for the respective data point. For each tilt angle we compute the mean deviation as $\chi^2 = \overline{\chi^2_{m_S}(t)}$, where the average is taken over all data points (in time) and the four spin-populations. This is plotted in Fig. 2 of the main text. We excluded $\theta = 0.2\pi$ since at this low angle there is not significant evolution of the population. We extract the overall best fitting B_{Q} from the averaged χ^2 over all tilt angles as $B_{\mathrm{Q}} \approx -3\,\mathrm{Hz}$. In the mean-field case, χ^2 deviates more wildly for large B_{Q}. The relatively best fit for all data points is found for $B_{\mathrm{Q}} \approx 1.1\,\mathrm{Hz}$. The overall deviation at the best value of B_{Q} is about three times smaller for the GDTWA than for the mean-field case [$\overline{\chi^2}_{\mathrm{GDTWA}} \approx 2.4$ and $\overline{\chi^2}_{\mathrm{MF}} \approx 7.3$, respectively]. Just for completeness in Supplementary Figure 2 we also show the GDTWA dynamics at the best possible positive $B_{\mathrm{Q}} = 6.3\,\mathrm{Hz}$, the value at which the second minimum in χ^2 is obtained (here, $\overline{\chi^2}_{\mathrm{GDTWA}} \approx 3.5$). Other parameters are identical to Fig. 2a in the main text. Also for this positive value of B_{Q} the agreement with the experiment is decent, however, some qualitative oscillations from the simulations are not perfectly reproduced by the experiment.

For the different long-time data set used for Fig. 3 of the main text, we perform the identical χ^2 optimization using an average taken over all available 7 Zeeman sub-states but for the shown

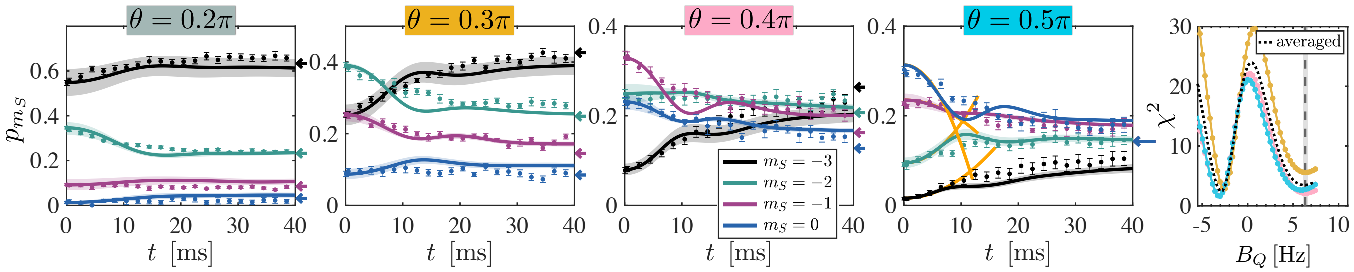

Supplementary Figure 2: Comparison of experimental data with GDTWA simulations. Identical two Fig. 2a of the main article, but instead of the best fitting B_{Q} value, the best fitting positive value of B_{Q} is chosen. We plot the four lowest spin-level populations, p_{m_S}, for various initial tilting angles $\theta = 0.2\pi$, 0.3π, 0.4π and 0.5π, on a $7 \times 3 \times 7$ cluster allowing the quadratic Zeeman field B_{Q} to be the only fitting parameter [here: $B_{\mathrm{Q}} = 6.3$Hz]. The red solid line (for $\theta = 0.5\pi$) is the result of the perturbative expansion, Eq. (3). The shaded area indicates the range of variation of the populations for evolutions with $\Delta B_{\mathrm{Q}} = \pm 0.3\,$Hz and uncertainties in the tilting angles with $\theta = (0.2 \pm 0.018\pi)$, $(0.3 \pm 0.012)\pi$, $(0.4 \pm 0.012)\pi$, $(0.5 \pm 0.01)\pi$ (estimated from the experiment). Error bars correspond to statistical standard deviations.

$\theta = 0.5\pi$ data only. For this different data set we find an optimal $\overline{\chi^2}_{\mathrm{GDTWA}} \approx 1.2$ for $B_{\mathrm{Q}} = -3.6\,$Hz, compared to a classical best fit with $\overline{\chi^2}_{\mathrm{MF}} \approx 2.9$ for $B_{\mathrm{Q}} = 1.1\,$Hz.

Supplementary Note 3

Dynamics of upper Zeeman sublevels not shown in main text

In Supplementary Figure 3 we show the four highest spin-level populations for various initial tilting angles $\theta = 0.2\pi$, 0.3π, 0.4π and 0.5π. They were omitted in the main text to make the figure less crowded.

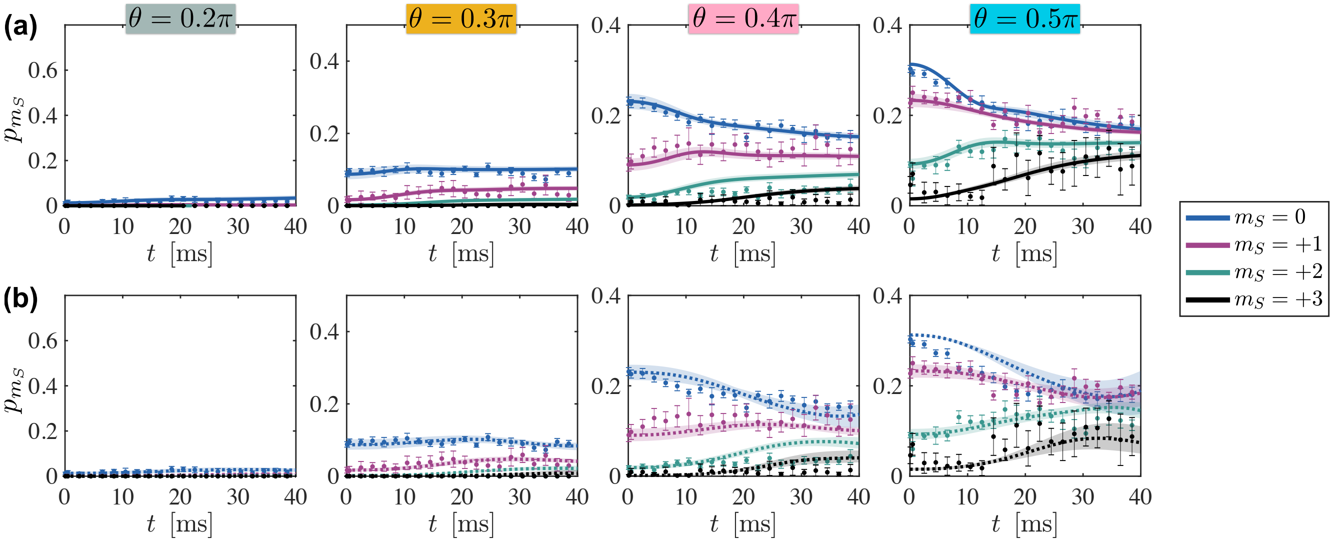

Supplementary Figure 3: Comparison between the quantum and classical dynamics for upper spin levels. We plot the spin-level populations, p_{m_S}, for various initial tilting angles $\theta = 0.2\pi$, 0.3π, 0.4π and 0.5π. Same panels as in Fig. 2 of the main manuscript, but for the spin levels $m = 0, +1, +2, +3$: (a) Comparison with GDTWA simulations (solid lines) on a $7 \times 3 \times 7$ cluster allowing the quadratic Zeeman field B_{Q} to be the only fitting parameter [here: $B_{\mathrm{Q}} = -3.0\,\mathrm{Hz}$]. (b) Comparison with the classical mean-field results (dotted lines) [here: $B_{\mathrm{Q}} = 1.1\,\mathrm{Hz}$]. Error bars correspond to statistical standard deviations.

Supplementary Note 4

System size convergence in numerical simulations Even with the classical equations it is very hard to model the macroscopic number of 10^4 atoms in the experiment. We therefore simulate the population dynamics of a bulk of atoms by using a small 3D $L_x \times L_y \times L_z$ block of increasing size and checking for finite size convergence. Due to the lattice geometry (with increased spacing in the y direction) finite size convergence is achieved for values $L_y < L_x, L_z$. Our finite size comparisons are summarized in Supplementary Figure 4 for both our GDTWA and mean-field simulations. In the GDTWA case it is evident that a size of $L_x \times L_y \times L_z = 6 \times 3 \times 6$ already give well converged results for all populations and all tilt angles on our time-scale of interest (for the results in the main text we use a $7 \times 3 \times 7$ block).

The mean-field result requires much larger system sizes for finite size convergence. This is a direct consequence of the different thermalization mechanism of the mean-field dynamics. Individual spin-density matrices remain pure in the mean-field case throughout the simulation. Finite entropy only builds up in the system-averaged local spin-density matrix, and stems from an inhomogeneous evolution of local phases due to field-gradients and finite-size effects. Therefore large system sizes are required for a converged average. In the main text, we use a system size of $L_x \times L_y \times L_z = 13 \times 4 \times 13$, which provides converged results on our time-scale of interest [cf. Supplementary Figure 4 (b)].

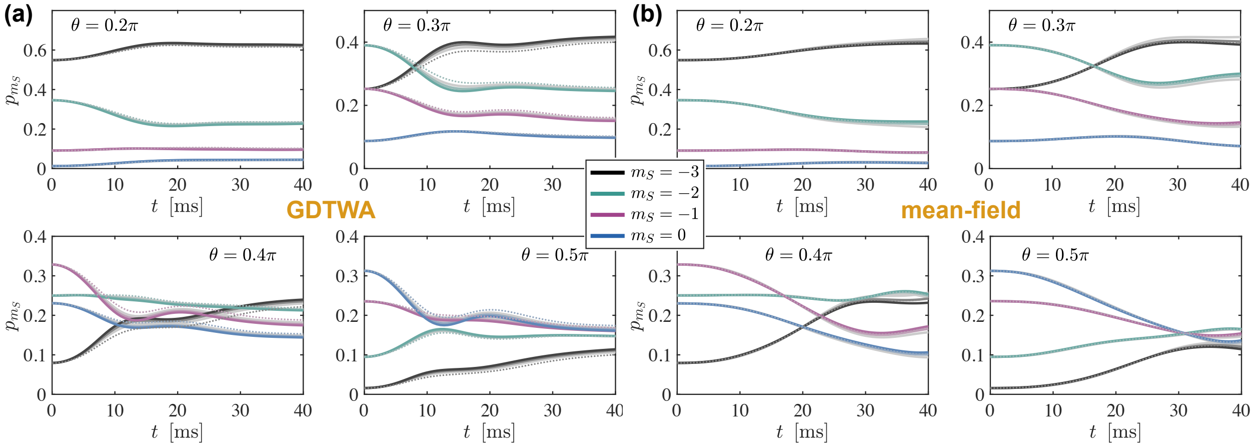

Supplementary Figure 4: System size convergence: time evolution of state populations for all four different initial tilt angles for the parameters used in the main text. The 3D system size $L_x \times L_y \times L_z$ is increased. (a) Results for GDTWA simualtions ($B_{\mathrm{Q}} = 4.4\,\mathrm{Hz}$). Three lines from light to dark color are for $L_y = 3$, and $L_x = L_z = 4, 5, 6$. The dashed lines are for a $4 \times 4 \times 4$ system and the overlap of the dashed line with the $4 \times 3 \times 4$ demonstrates convergence in the y direction. (b) Same results for mean-field simulations ($B_{\mathrm{Q}} = 1.4\,\mathrm{Hz}$), light to dark lines are for $L_y = 4$, and $L_x = L_z = 9, 11, 13$, and dashed lines are for a $11 \times 3 \times 11$ system.

Supplementary Note 5

Effect of magnetic field gradients on spin dynamics We have investigated the effect of magnetic field gradients on spin dynamics predicted by GDTWA. This effect is very small at short times (as expected from perturbation theory, see Eq. (3) of the main text), and only slightly inhibits dynamics at longer times, as shown in Supplementary Figure 5.

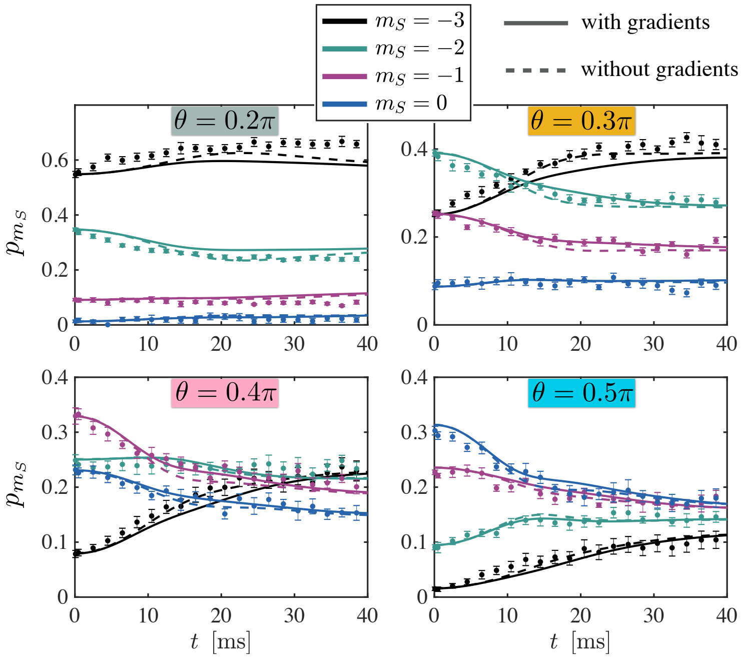

Supplementary Figure 5: Effect of the magnetic field gradient on GDTWA simulations. We show experimental data (error bars correspond to statistical standard deviations), and GDTWA simulations for the populations with identical parameters as in Fig. 2 of the main manuscript. Dashed lines show results for zero field gradient. The solid line is the result with the measured field gradient of 30 MHz.m^{-1} along the vertical axis. A magnetic gradient slightly inhibits dynamics, after $\sim 10\,$ms.

Supplementary Note 6

Effect of non-ideal preparation on spin dynamics We have investigated the effect of defects due to imperfect preparation on spin dynamics predicted by GDTWA. The presence of 10% hole defects barely modifies the dynamics, as shown in Supplementary Figure 6.

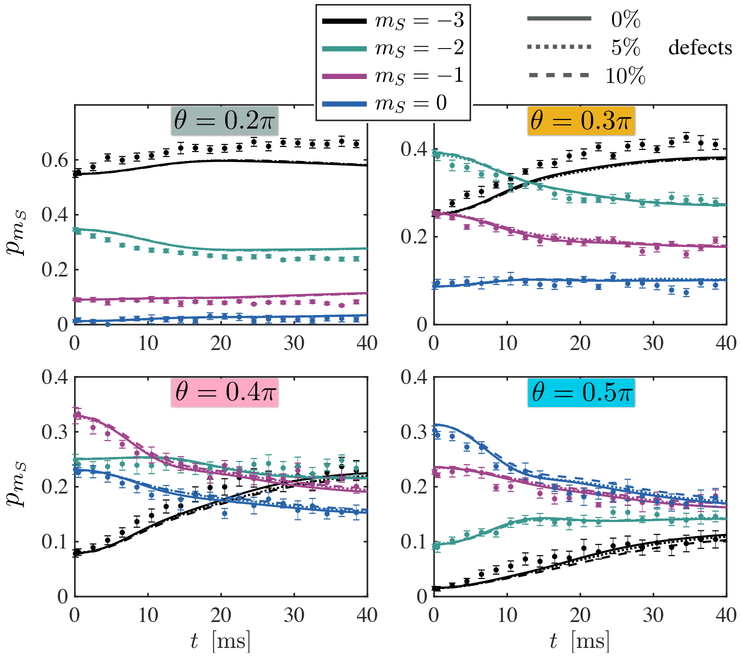

Supplementary Figure 6: Effect of lattice defects on GDTWA simulations. We show experimental data (error bars correspond to statistical standard deviations), and GDTWA simulations (full lines) for the populations with identical parameters as in Fig. 2 of the main manuscript. Additionally, dotted and dashed lines show results for 5% and 10% defect density, respectively. The statistical average over 192 different defect realizations has been taken into account. Differences are more pronounced for larger tilting angles, but still minor.

Supplementary Note 7

Long-time dynamics and quantum thermalization In the main article (see Figure 3) we discuss about the thermalization process occurring in our spin system. We derive an analytic formula which links the long time populations of the different Zeeman levels to the ones of a thermal ensemble with the same energy and magnetization of the initial state. The formula, however, is not exact. It is based on a high temperature perturbative expansion which is strictly valid in the regime $B_{\mathrm{Q}} \ll \sqrt{V_{\mathrm{eff}}}$. In this section we benchmark the validity of the formula by comparisons with GDTWA dynamics at different quadratic fields. The summary is presented in Supplementary Figure 7 (a)-(c), where we show that the analytic formula is valid for the experimental range of B_{Q} parameters.

In addition to the quadratic Zeeman field, magnetic field gradients are also present in the experiment. While those terms can affect the thermalization dynamics leading to the development of spatial magnetization textures at equilibrium, we argue in the main text that such a texture is expected to occur at extremely long times in a dipolar interacting system since it requires interactions between remote parts of the cloud. Here we validate such claim using GDTWA simulations. As seen in Supplementary Figure 7 (d) the gradients flatten out the population distribution compared to the zero gradient case, but this effect requires very large timescale compared to those currently accessible in the experiment.

In Supplementary Figure 8, we show all available long time data for the asymptotic spin populations obtained under different experimental measurements. As the quadratic light shift B_{Q} is

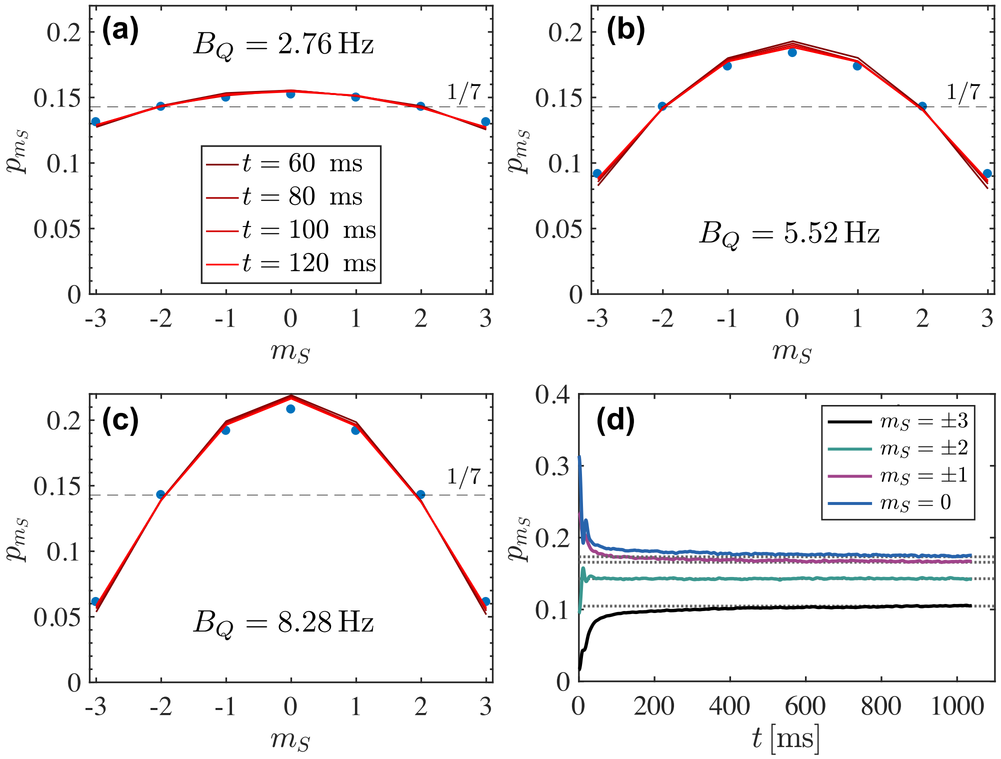

Supplementary Figure 7: GDTWA vs. analytical formula: (a)-(c) Comparisons of various late time Zeeman level populations for zero magnetic field gradients and different quadratic tensor light shifts $B_\mathrm{Q} = 2.76\,\mathrm{Hz}$, $B_\mathrm{Q} = 5.52\,\mathrm{Hz}$, and $B_\mathrm{Q} = 8.28\,\mathrm{Hz}$, respectively ($7 \times 3 \times 7$ lattice). The blue points show the analytical thermalization values from Eq. (6). GDTWA results converge to the thermal prediction for all the B_Q cases displayed. (d) Long-time evolution up to $1\,\mathrm{s}$ with half the field-gradients from the experiment (and $B_\mathrm{Q} = 5.52\,\mathrm{Hz}$). Horizontal dotted lines show the prediction from Eq. (6) after including the exact gradients on the $7 \times 3 \times 7$ block. At the experimentally relevant time-scales ($\sim$ 80ms), the population redistribution stems mostly from the interactions and the quadratic tensor light shifts. Only on a much longer scale ($\sim$ 0.5s) the redistribution from the field gradients causes a relaxation of the GDTWA simulation to the analytical estimation including gradients.

very sensitive to the relative intensity of the five different lattice laser beams, we expect variations of $B_{\rm Q}$ between different data sets and therefore, accordingly to the analytic formula, a correspondent variation on the fractional populations p_{m_S} at long times, except from $p_{\pm 2}$ which should remain close to $1/7$. All these features are indeed observed in Supplementary Figure 8.

We use the model to find the value of $B_{\rm Q}$ that best fits the data, from which the temperature T at which thermalization takes place can be inferred through Eq.(6). We point out that, similar to the case shown in Fig 2 of the main paper, comparing our data to the analytical model typically leads to two possible values of $B_{\rm Q}$ (one positive, and one negative), for which the χ^2 shows a local minimum. Distinguishing between these two local minima is difficult (i.e. they share a similar χ^2) which is a consequence that $|B_{\rm Q}| > |\bar{V}|$.

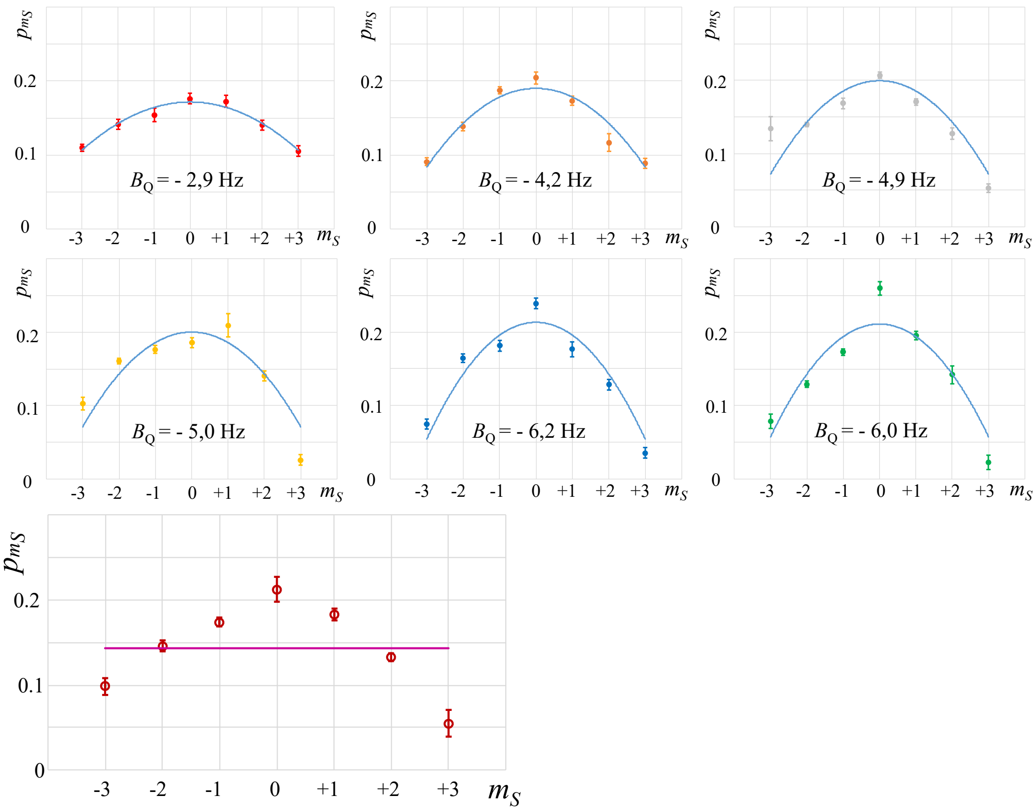

Supplementary Figure 8: Thermalization at long time: extensive data, and comparison with our analytical model. Top 6 figures (filled circles): long time spin populations for 6 different data sets. The full lines correspond to the result of the analytical model using B_{Q} as a fitting parameter, whose (negative) value is displayed. Error bars correspond to statistical standard deviations. Bottom: the empty circles correspond to average of these 6 data, the error bar corresponding to the associated standard deviation. The full line indicates 1/7.
